# Association Between Self-Rated Health and Medical Care Disruption Due to COVID-19 Among Individuals With Atherosclerotic Cardiovascular Disease

**DOI:** 10.7759/cureus.40697

**Published:** 2023-06-20

**Authors:** Akbar Hussain, Okelue E Okobi, Chinedum B Obi, Vivian C Chukwuedozie, Cherechi G Sike, Eghogho H Etomi, Falilatu B Akinyemi

**Affiliations:** 1 Internal Medicine, Appalachian Regional Health, Harlan, USA; 2 Family Medicine, Medficient Health Systems, Laurel, USA; 3 Family Medicine, Lakeside Medical Center, Belle Glade, USA; 4 Internal Medicine, Imo State University, Owerri, NGA; 5 Internal Medicine, Ebonyi State University Medical School, Abakaliki, NGA; 6 General Practice, Windsor University School of Medicine, Cayon, KNA; 7 Cardiology, Kharkiv National Medical University, Kharkiv, UKR; 8 Research, Texas Southern University, Houston, USA; 9 Internal Medicine, Windsor University School of Medicine, Cayon, KNA

**Keywords:** ascvd, medical care, atherosclerotic cardiovascular disease, covid-19, self-rated health

## Abstract

Background: The coronavirus disease 2019 (COVID-19) pandemic has disrupted medical care across diverse populations with varying outcomes. In this study, we evaluated the relationship between health rating and disruption in medical care due to COVID-19 among individuals with atherosclerotic cardiovascular disease (ASCVD).

Methods: Data from the 2020 National Health Interview Survey was used for this study. ASCVD sample included those with self-reported coronary heart disease, stroke, and heart attack. Health rating was represented as fair to poor, good, and very good to excellent. The outcome variable was a disruption of medical care due to COVID-19 (delay in medical care or did not get care). The chi-square test was used for the descriptive analysis and multiple logistic regression was used to evaluate the relationship between health rating and disruption in medical care with demographic factors, comorbidities, and cumulative social risk adjusted for.

Results: Among the 31,568 adults, 1,707/31,568 representing 9,385,855 adults 18 years and above with ASCVD reported experiencing or not experiencing a disruption in medical care. After adjusting for cumulative risk, the odds of not getting medical care due to COVID-19 were high for those who rated their health as fair/poor as compared to excellent (adjusted odds ratio (AOR) = 1.95, 95% CI = 1.24-3.08, p = 0.004). These odds remained about the same after adjusting for cumulative social risk, demographic factors, and comorbidities (AOR = 1.84, 95% CI = 1.11-3.06, p = 0.018). After adjusting for cumulative risk, medical care utilization (received, delayed, did not receive) was rated. Those who rated their health as fair to poor as compared to excellent were more likely to report a delay in health care due to COVID-19 (AOR = 1.85, 95% CI = 1.28-2.68, p = 0.001) and remained about the same after adjusting for cumulative social risk, demographic factors, and comorbidities (AOR = 1.86, 95% CI = 1.22-2.82, p = 0.004). Female respondents with ASCVD who rated their health as fair/poor were more likely to experience a delay in medical care due to COVID-19 (AOR = 2.06, 95% CI = 1.06-4.01, p = 0.033) or not get medical care due to COVID-19 (AOR = 2.86, 95% CI = 1.42-5.76, p = 0.003) as compared to those who rated their health as excellent. With regards to men with ASCVD, health rating was not related to their reported disruption of medical care due to COVID-19.

Conclusions: A poor to fair health rating is associated with a delay in getting or not getting medical care among individuals with ASCVD. Further studies are needed to evaluate this relationship further.

## Introduction

Despite the decrease in atherosclerotic cardiovascular-related mortality within the past half century, it remains the leading cause of death in the United States (US) [1]. In recent times, this burden has been further compounded by the disruption of medical care by the coronavirus disease 2019 (COVID-19) pandemic. In 2020, the Centers for Disease Control and Prevention estimated that 41% of US adults had delayed or avoided medical care [2]. The main reasons for the disruption of medical care are health system-related and patient factors such as gender, level of education, and health perception [3,4]. This disruption of medical care has been associated with negative outcomes in individuals with heart failure and those who experience an out-of-hospital cardiac arrest in the US [5,6]. The relationship between self-rated health, which is a measure of health perception, and medical care disruption among adults with ASCVD has, however, not been studied using a nationally representative sample.

Self-rated health is a measure of general health [7]. It is influenced by physical and socioeconomic factors among other predictors [8] and has been identified as an excellent risk indicator for atherosclerotic cardiovascular disease (ASCVD) outcomes [9]. In a large multiethnic US study, self-rated health was shown to complement and even enhance coronary artery calcium score in its risk prediction of cardiovascular outcomes [9]. Further, COVID-19 has been acknowledged to bring about cardiac involvement at the microvascular and macrovascular levels in addition to causing an array of cardiac complications and electrocardiographic abnormalities [10-12]. In addition, self-rated health was reported as a significant associate of incident ischemic heart disease [10] and major cardiovascular events in populations with stable coronary heart disease [11]. Hence, self-rated health may not only contribute to medical care disruption but may also enhance markers of increased risk in populations with ASCVD.

Given the reported medical care disruption across diverse populations due to the COVID-19 pandemic, the potential of self-rated health to act as a modifier of health-seeking behavior and as well act as a risk indicator for ASCVD, it is important to study the effect of self-rated health on medical care disruption due to the COVID-19 pandemic using a nationally representative sample of adults with ASCVD. In this study, we evaluated the relationship between health rating and disruption in medical care due to COVID-19 among individuals with ASCVD considering the social determinants of health and assessed the relationship between gender and medical care disruption by health rating status.

The current research aimed to evaluate the relationship between self-reported health ratings among populations affected by ASCVD and disruption in medical care due to COVID-19. Further, an assessment of the relationship between gender and medical care disruption by health-rated status was conducted. We believe using a nationally representative sample to assess medical care disruption due to COVID-19 is valuable due to the wide-scale disruption of medical services during the COVID-19 pandemic and the consequent adverse outcomes, especially in women [13,14]. To our knowledge, this work is the first to assess medical disruption among individuals with ASCVD with a subanalysis by gender. The findings from this study demonstrate that some adverse cardiovascular outcomes of the COVID-19 pandemic may be due to medical care disruption among populations with ASCVD.

## Materials and methods

Data source

This study uses data from the 2020 National Health Interview Survey (NHIS). The NHIS is a cross-sectional household interview survey. It is a source of information on the health of the non-institutionalized civilian population of the United States conducted yearly by the Centers for Disease Control and Prevention’s National Center for Health Statistics (NCHS). This survey is used to monitor the health of the US population through the collection of a broad range of health topics such as self-reported health rating, demographic and socioeconomic characteristics, comorbid medical conditions, and more recently the impact of COVID-19 on medical care. Due to the COVID-19 pandemic, data collection was via telephone and in-person visits. The sample adult data with a response rate of 48.9% was used for this analysis. This sample adult data contain information from 31,568 sample adult interviews. More information about the NHIS is available online. In this research, publicly accessible secondary data from the NHIS database were analyzed. Since analyzing publicly available datasets typically does not necessitate IRB approval, this study did not require an IRB approval number, following ethical research guidelines. The publicly available data used in this study do not contain personally identifiable information and do not involve human subjects.

Study design

This was a cross-sectional study of adults 18 years and above who reported having ASCVD. ASCVD status was determined from the questions “ever been told you had coronary heart disease, ever been told you had a heart attack, and ever been told you had a stroke.” Those who reported yes to these questions were classified as having ASCVD. Furthermore, the study assessed satisfaction with medical care among patients with ASCVD during the COVID-19 pandemic, considering the categories of delayed, received, or never received care.

Study population

Among the 31,568 adults, 76 of them did not respond to the questions on ASCVD and were considered as missing, which made up <10% of the population and was not taken into account in the analysis. Among the population, 1,707/31,568 adults with ASCVD representing 9,385,855 US adults 18 years and above responded to the question on delays in medical care due to COVID-19.

Sociodemographic variables

The variables of interest were age, sex, race, marital status, level of education, income, employment status, type of insurance, income from others, settlement, and food insecurity. They were represented as age (≤40, 41-64, and 65 years and above), sex (female and male), race (non-Hispanic (NH) white, NH black or African Americans, NH Asian, and Hispanic), marital status (never, married, and formerly married), level of education (no college degree, some college, and college degree), income (<$50,000, $50,000-99,999, and $100,000), employment status (unemployed and employed), insurance type (uninsured, private, and Medicaid insurance), income from others (no support and support from other sources), settlement (rural, suburban, and urban), and food insecurity (never, sometimes, and often true). Like the method reported by Hagan et al. [12], a cumulative variable to denote cumulative social risk was developed from the combination of seven variables, which were education, settlement, insurance, food insecurity, income, income from others, and employment. From the composite value generated, four quartiles were created from quartiles 1 to 4 with quartile 4 denoting the highest cumulative social risk and quartile 1 denoting the lowest risk.

Comorbid variables

These include BMI, smoking status, diabetic mellitus, hypertension, renal disease, chronic obstructive pulmonary disease (COPD), and liver cirrhosis. These were represented as BMI (non-obese and obese), smoking status (never, former, and current), diabetic mellitus (non-diabetic and diabetic), hypertension (non-hypertensive and hypertensive), renal disease (no renal disease and renal disease), COPD (no COPD and COPD), and liver cirrhosis (no liver cirrhosis and liver cirrhosis).

Outcome variable

The outcome variable included disruption of medical care due to COVID-19. It was identified from reports of yes or no to “delayed medical care due to COVID-19 and did not get medical care due to COVID-19.” In this study, those who reported yes were said to have experienced a disruption of medical care due to COVID-19.

Statistical analysis

Sample weight was applied during the analysis due to the complex design of the study and to account for the response rate. Descriptive analysis was used to assess the distribution of the respondents with ASCVD by reported disruptions in medical care due to COVID-19 with observed frequencies and weighted proportions reported. A bivariate analysis was used to show the weighted percentages of respondents with ASCVD who experienced a disruption of medical care due to COVID-19 by health rating status. Multiple logistic regression was used to show the unadjusted and adjusted odds of experiencing a disruption in medical care to COVID-19 among respondents with ASCVD, with cumulative social risk adjusted for in the second model and cumulative social risk, demographic characteristics, and comorbidities adjusted for in the third model. We further evaluated gender differences in the odds of experiencing a disruption of medical care by those with ASCVD who rated their health as fair with a logistic regression model with an adjusted odds ratio reported after adjusting for cumulative social risk, demographic factors, and comorbid features. STATA 14.0 (StataCorp LLC, College Station, TX) was used for this analysis with the p-value set at <0.05 for all two-tailed tests.

## Results

Table 1 below shows the baseline characteristics of the study population. Among those with ASCVD who reported not getting medical care due to COVID-19, they were mostly females as compared to males (24.1% vs. 17.9%), had a college degree as compared to some or no college degree (26.6% vs. 20.5% vs. 17.6%), and were diabetics (27.9% vs. 17.6%). Among those who reported delaying medical care due to COVID-19, they were mostly 41 to 64 years old (35.9%) as compared to those aged 40 years (26.4%) and 65+ years (24.0%), females as compared to males (34.3% vs. 26.3%), and college graduates (38.8%) as compared to some college (27.5%) or no college attendance (26.7%). The rest of the distribution is as shown below.

**Table 1:** Baseline characteristics of respondents with ASCVD who experienced a disruption of medical care due to COVID-19. ASCVD: atherosclerotic cardiovascular disease; BMI: body mass index; COPD: chronic obstructive pulmonary disease; NH: non-Hispanic; (-): cells intentionally left empty. Please note that these p-values indicate the level of significance for rejecting the null hypothesis of independence. A p-value less than 0.05 is typically considered statistically significant, suggesting that the variables are not homogeneously distributed among the groups.

	Reported that they did not get care due to COVID-19, n (weighted %)	Reported no delay in medical care due to COVID-19, n (weighted %)	P-value
Age: years <40, 41-64, >65	10 (22.8%), 121 (24.6%), 213 (17.9%)	11 (24.0%), 174 (35.9%), 331 (26.4%)	0.12
Sex: female, male	164 (24.1%), 180 (17.9%)	246 (34.3%), 270 (26.3%)	0.001
Race: NH white, NH black, NH Asian, Hispanic	265 (20.2%), 38 (21.7%), 23 (22.6%), 8 (18.0%)	410 (30.0%), 46 (25.1%), 35 (32.3%), 9 (28.4%)	0.113
Marital status: never married, married, formerly married	69 (27.7%), 133 (17.8%), 142 (20.6%)	101 (34.4%), 210 (28.1%), 205 (29.3%)	0.001
Level of education: less than college, some college, college degree	115 (17.6%), 105 (20.5%), 121 (26.6%)	177 (26.7%), 157 (27.5%), 178 (38.8%)	0.001
Employment status: unemployed, employed	253 (20.0%), 82 (22.1%)	385 (29.5%), 121 (32.1%)	0.001
BMI: not obese, obese	202 (20.1%), 137 (20.7%)	297 (27.9%), 213 (32.1%)	0.001
Smoking status: never smoked, current smoker, former smoker	166 (22.9%), 49 (20.9%), 123 (17.6%)	234 (31.5%), 71 (34.5%), 205 (26.9%)	0.001
Income level:	192 (20.1%), 88 (19.1%), 64 (23.5%)	278 (27.9%), 138 (30.8%), 100 (32.6%)	0.002
Insurance status: uninsured, private, Medicaid	7 (15.6%), 152 (20.8%), 184 (20.3%)	12 (23.0%), 228 (30.9%), 275 (29.3%)	0.001
Diabetic mellitus status: non-diabetic, diabetic	231 (17.6%), 113 (27.9%)	360 (28.1%), 155 (33.4%)	0.001
Hypertension: no hypertension, hypertension	94 (22.0%), 250 (19.9%)	137 (28.8%), 379 (29.9%)	0.001
Renal disease: no renal disease, renal disease	297 (20.2%), 46 (21.5%)	449 (29.0%), 66 (33.1%)	0.001
COPD status: no COPD, COPD	265 (19.9%), 78 (23.0%)	394 (28.6%), 119 (34.0%)	0.001
Liver cirrhosis: no liver cirrhosis, liver cirrhosis	332 (20.2%), 10 (29.6%)	496 (29.4%), 18 (34.6%)	0.001

**Figure 1:**
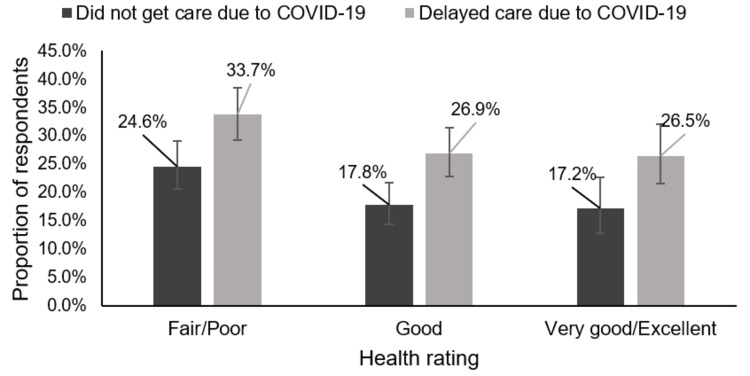
Health rating distribution of study respondents who had medical care disruption due to COVID-19.

In this ASCVD population, 570/1,707 (32.7%) reported either a delay in medical care due to COVID-19 or that they did not get care due to COVID-19. Figure 1 below shows the health rating distribution of the study respondents by their experience of disrupted medical care due to COVID-19. Among those who did not get care due to COVID-19, the proportion of respondents with ASCVD reduced as the health rating improved from fair/poor (24.6% (20.6-29.1)) to good (17.8% (14.4-21.7)) and then very good/excellent (17.2% (12.8-22.6)). A similar trend was seen among those who had a delay in medical care due to COVID-19. The proportion reduced as the health rating improved from fair/poor (33.7% (29.2-38.5)) to good (26.9% (22.8-31.4)) and to very good/excellent (26.5% (21.5-32.1)).

**Figure 2:**
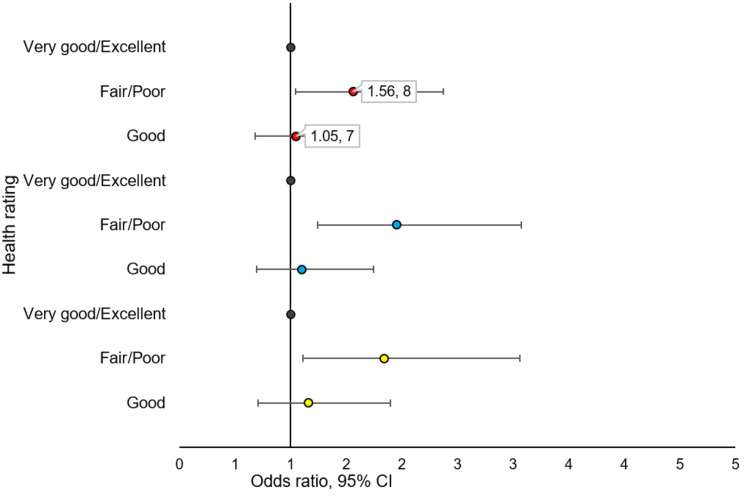
Unadjusted and adjusted odds of not getting care due to COVID-19 among respondents with ASCVD. Orange: unadjusted model; blue: adjusted for cumulative social disadvantage; yellow: adjusted for cumulative social disadvantage, demographic characteristics, and comorbidities. ASCVD: atherosclerotic cardiovascular disease.

Figure 2 below shows the unadjusted and adjusted odds of not getting care due to COVID-19 among respondents with ASCVD. In the unadjusted model, as compared to those who rated their health as excellent, those who rated their health as fair/poor had an increased odd of not getting care due to COVID-19 (OR = 1.57, 95% CI = 1.05-2.37, p = 0.029). After adjusting for cumulative risks, the odds of not getting medical care due to COVID-19 increased for those who rated their health as fair/poor as compared to excellent (adjusted odds ratio (AOR) = 1.95, 95% CI = 1.24-3.08, p = 0.004). This odd remained about the same after adjusting for cumulative social risk, demographic factors, and comorbidities (AOR = 1.84, 95% CI = 1.11-3.06, p = 0.018). However, among those who rated their health as good as compared to excellent health rating, there was no significant association in the unadjusted or adjusted models with regards to not getting medical care due to COVID-19.

**Figure 3:**
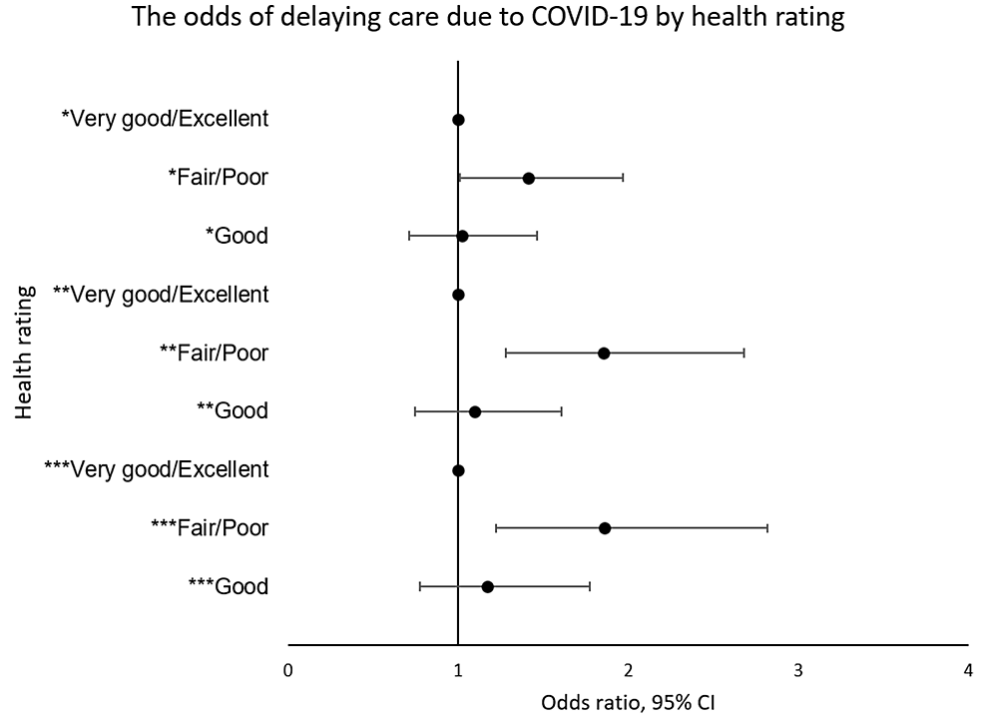
Unadjusted and adjusted odds of experiencing a delay in medical care due to COVID-19. * Unadjusted model. ** Adjusted for cumulative social disadvantage. *** Adjusted for cumulative social disadvantage, demographic characteristics, and comorbidities.

Figure 3 below shows the result of unadjusted and two adjusted models. In the unadjusted model, a fair/poor health rating as compared to an excellent health rating was associated with a delay in health care due to COVID-19 (OR = 1.41, 95% CI = 1.01-1.97, p = 0.042). This odd increased after adjusting for cumulative social risk (AOR = 1.85, 95% CI = 1.28-2.68, p = 0.001) and remained about the same after adjusting for cumulative social risk, demographic factors, and comorbidities (AOR = 1.86, 95% CI = 1.22-2.82, p = 0.004). However, a good health rating as compared to an excellent health rating is not associated with a delay in health care due to COVID-19 (OR = 1.02, 95% CI = 0.71-1.47, p = 0.904). This relationship remained after adjusting for cumulative social risk (AOR = 1.10, 95% CI = 0.75-1.61, p = 0.634), and cumulative social risk, demographic factors, and comorbidities (AOR = 1.17, 95% CI = 0.78-1.77, p = 0.448).

Tables 2, 3 show the adjusted odds of experiencing either a delay in medical care due to COVID-19 or not getting care due to COVID-19. After adjusting for demographic factors, comorbidities, and cumulative social risk, female respondents with ASCVD who rated their health as fair/poor were more likely to experience a delay in medical care due to COVID-19 (AOR = 2.06, 95% CI = 1.06-4.01, p = 0.033) or not get medical care due to COVID-19 (AOR = 2.86, 95% CI = 1.42-5.76, p = 0.003) as compared to those who rated their health as excellent. With regards to men with ASCVD, health rating was not related to their reported disruption of medical care due to COVID-19 with cumulative social risk, demographic factors, and comorbidities adjusted for.

**Table 2:** Male respondents with different health rating and their odds of experiencing a disruption in medical care due to COVID-19. AOR: adjusted odds ratio; (-): no value required.

	Delayed medical care due to COVID-19		Did not get medical care due to COVID-19	
Health rating	AOR, 95% CI	p-value	AOR, 95% CI	p-value
Excellent	1.00	-	1.00	-
Fair/poor	1.48 (0.84-2.59)	0.172	1.35 (0.70-2.59)	0.365
Good	1.16 (0.69-1.96)	0.567	0.96 (0.50-1.82)	0.893

**Table 3:** Female respondents with different health rating and their odds of experiencing a disruption in medical care due to COVID-19. AOR: adjusted odds ratio; (-): no value required.

	Delayed medical care due to COVID-19		Did not get medical care due to COVID-19	
Health rating	AOR, 95% CI	p-value	AOR, 95% CI	p-value
Excellent	1.00		1.00	-
Fair/poor	2.06 (1.06-4.01)	0.033	2.86 (1.42-5.76)	0.003
Good	1.10 (0.57-2.12)	0.779	1.56 (0.76-3.23)	0.225

## Discussion

In this nationally representative study of the US population with ASCVD in 2020, 32.7% reported either a delay in medical care due to COVID-19 or that they did not get care due to COVID-19. This proportion is slightly lower when compared to the proportion of general US adults (40.9%) who reported a delay or avoidance of medical care due to concerns about COVID-19 [2]. It is, however, similar to the proportion (33.6%) reported from a 2020 internet-based study of US adults [15]. Even after age group stratification, the proportions of US younger and older adults who reported experiencing delays in medical care due to the COVID-19 pandemic remained similar to ASCVD populations in our study who reported these delays [2,16,17]. These disruptions in medical care among adults with ASCVD may be responsible for some of the reported healthcare usage and outcomes seen during the COVID-19 pandemic, as studies have shown a decrease in acute heart failure hospitalizations [18], emergency room presentation of acute ischemic stroke patients [19], and a reduction in ST-segment myocardial infarction (STEMI) admissions [20] among ASCVD populations. Given that individuals with ASCVD who rate their health poorly are less likely to seek medical care, as stated earlier, we went on to evaluate if a similar trend persisted during the COVID-19 pandemic.

In evaluating the relationship between health rating and medical care disruptions, we observed that among the individuals with ASCVD, as compared to those with an “excellent” health-rated status, a “fair” or “poor” health-rated status was associated with both a delay in medical care and not getting medical care due to COVID-19. This relationship, however, was not evident when comparing a “good” health rating to a “very good” or “excellent” health rating. With the emergence of the COVID-19 pandemic, fair or poor general health was reportedly associated with a delay in medical care among older US adults [21], an observation also reported by Zhong et al. [16]. Studies in the pre-COVID-19 pandemic era demonstrated a similar relationship between poor self-rated health and medical care disruption. In a prospective study of Finnish adults, Miilunpalo et al. reported an inverse relationship between self-rated health and the number of physician contacts [22]. Similarly, Giannouchos et al. reported an association between delayed medical care and worse self-reported health rating [17]. Although this ASCVD population did report a delay or not getting medical care due to COVID-19, this medical care disruption may be more related to their health status at the time of medical need and due to the COVID-19 pandemic.

A study by Callison and Ward that focused on reasons for medical care disruption due to COVID-19 reported that a greater level of education and having health insurance coverage were associated with a delay in medical care [23], factors that are generally associated with better socioeconomic status and good health literacy level. Similarly, our study’s population with medical care delays was mostly those with a college degree, higher income, and who reported employment status. Conversely, a study reported that factors such as difficulties in paying usual household expenses, Veteran Affairs or Medicaid, factors that are associated with a poor socioeconomic status, and poor health literacy were associated with delays in medical care due to COVID-19 [17]. A study by Ahn et al. further illuminated the relationship between the pandemic and the utilization of healthcare services [24]. The findings opt for a supportive approach to data interpretation for our research as care is not inaccessible due to the pandemic but is rather used less. It can be argued that this is just as significant in its impact as involuntary healthcare delays on the end of the providers. More specifically, patient visits decreased by 30% and out-of-pocket medical spending decreased by 23%; this resulted in the diagnoses of chronic conditions reduced by 19% in April 2020 [24]. The decrease in out-of-pocket spending implies the financial burden of care amplified by the pandemic, creating further reasoning for patient-oriented underutilization of care. Perhaps among ASCVD populations, socioeconomic factors that are related to self-rated health perception probably do play a role in medical care decisions among these individuals, and even in the presence of a pandemic, this relationship remains pervasive. The relationship between health rating and delayed care may also be a reflection of the COVID-19 threat that prevents patients from going in for an appointment. The findings can be further applied to the ASCVD population, as the risk of respiratory infection may be more prominent than in those with no existing condition.

Regarding gender differences in the relationship between health-rated status and delay in receiving medical care or not receiving medical care, a “fair” to “poor” health rating as compared to “very good” to “excellent” health was associated with increased odds of disruption of medical care in women with ASCVD but not in men. An article put forth by Okunrintemi et al. prior to the pandemic concluded that women with ASCVD as compared to men were more likely to experience lower healthcare satisfaction, poor perception of health status, and lower health-related quality of life [25]. With the emergence of the COVID-19 pandemic, an unfavorable gender gap in health care, as well as adverse outcomes among women as compared to men, was reported, with factors such as a lack of access to health care and low healthcare capacity being mentioned as reasons for this existing gap [26]. Furthermore, Pacheco et al., in evaluating access to care for time-sensitive conditions, reported a greater reduction in healthcare access in women as compared to men with cardiovascular diseases [27], and a similar finding was reported in non-ASCVD populations [28]. To understand the impacts of a pandemic-induced healthcare delay over a longer time frame, Gertz et al. reported that those with a higher risk of COVID-19, for example, women with a fair to poor health-rated status who experience delays in medical care, are more likely to exacerbate pre-existing chronic conditions leading to poorer long-term health [29-31]. Thus, efforts are needed to improve the health status of individuals with ASCVD, most especially women, as an existing gender gap in health care is most likely to worsen in the presence of circumstances that strain the health sector even more.

Strengths and limitations

This study has a few strengths worthy of note. Our study makes use of a nationally representative large sample size of adults in answering an important question that arose during the current COVID-19 pandemic. Considering the importance of the social determinants of health in health outcomes of individuals with ASCVD, we controlled for social risk in our logistic models to eliminate as much as possible the influence this has on self-rated health and medical care delays. A sub-analysis of gender differences was conducted to further evaluate the impact of self-rated health on medical care delays. The sub-analysis of gender differences was considered important, as it provided a deeper context into our findings due to the existing gender gap in healthcare disparity in individuals with ASCVD.

For the limitations of the study, as with every retrospective study using national survey data, there exists a potential for recall bias, as these decisions to delay care may have occurred months or weeks prior to when the survey interview was conducted. Self-reporting bias is another challenge as this is unaccounted for by race or demographic information, as some individuals are more prone to discomfort than others, resulting in a better health rating. From the survey data, it is difficult to fully understand why they delayed medical care due to COVID-19. It may have been due to efforts from respondents with non-severe COVID-19 infection to avoid a further spread of the virus, fear of contracting the COVID-19 virus, or for numerous other reasons that were not stated and as such leaves room for different interpretations. In a similar line of thought, a causal relationship is difficult to establish, not only due to the retrospective nature of the study but also due to the vagueness of the survey questions. This study only evaluated the short-term consequences of the COVID-19 pandemic, leaving a gap as to what happened to these respondents with ASCVD in the long term. Also, it is essential to consider the potential influence of recall bias during the data collection phase of NHIS. While this study analyzed NHIS data, it is important to acknowledge that recall bias could have impacted the outcomes, leading to potential implications for the results and conclusions.

## Conclusions

A significant proportion of individuals (32.7%) with ASCVD reported experiencing a disruption in medical care due to COVID-19, which was a similar proportion of the general US adult population who also reported a disruption in medical care. Findings presented here suggest fair/poor health-rated status to be one of them, with women (24.1%) more likely to report this medical care disruption as compared to men (17.9%) during the COVID-19 pandemic. Although the socio-determinants of health that impact self-rated health were controlled for in these findings, their direct relationship with self-rated health cannot be fully accounted for in a regression model. Among patients with ASCVD, efforts are needed to address factors that negatively impact self-rated health status, as a significant strain on the healthcare sector, such as the emergence of a pandemic, can further worsen the existing healthcare gap in the US. Further studies are needed to evaluate the short and long-term impact of medical care disruption among adults with ASCVD. This is due to the potential for chronic medical conditions to worsen in the presence of a pandemic by worsening self-rated health and creating a negative feedback loop.
